# Reproduction of Bone from Periosteum in Resection of Joints

**Published:** 1869-09

**Authors:** 


Reproduction of Bone from Periosteum in Resection of Joints.—Experiments made by M. Ollier, of Lyons, conclusively establish the power of periosteum to reproduce bone. These experiments are of great importance as bearing on resection of joints. (p. 132.)

When the elbow joint is excised, the bones only being removed, and the periosteum left entire, it is a most singular and startling fact that reproduction of the bone takes place, the form of the original bone being preserved. A case is related in which both the condyles of the humerous and the olecranon process were reproduced. There was a regular joint, surrounded by a capsule and containing hyaline cartilage. The details of the operation are described. (Mr. T. Holmes, p. 129.)

